# The Effect of Eu Doping on Microstructure, Morphology and Methanal-Sensing Performance of Highly Ordered SnO_2_ Nanorods Array

**DOI:** 10.3390/nano7120410

**Published:** 2017-11-23

**Authors:** Yanping Zhao, Yuehua Li, Xingping Ren, Fan Gao, Heyun Zhao

**Affiliations:** 1 College of Materials Science and Engineering, Yunnan University, Kunming 650091, China; skyzyp@foxmail.com (Y.Z.); f_gao_yn@sohu.com (F.G.); 2 Advanced Measurement and Analysis Center of Dali University, Dali 671200, China; yuehua_li99@sohu.com; 3 Yunnan Security and Technology Co. Ltd., Kunming 650033, China; 4 Yunnan Key Laboratory for Micro/Nano Materials and Technology, Yunnan University, Kunming 650091, China

**Keywords:** tin dioxide, nanorods arrays, Eu dopant, hydrothermal route, gas sensor

## Abstract

Layered Eu-doped SnO_2_ ordered nanoarrays constructed by nanorods with 10 nm diameters and several hundred nanometers length were synthesized by a substrate-free hydrothermal route using alcohol and water mixed solvent of sodium stannate and sodium hydroxide at 200 °C. The Eu dopant acted as a crystal growth inhibitor to prevent the SnO_2_ nanorods growth up, resulting in tenuous SnO_2_ nanorods ordered arrays. The X-ray diffraction (XRD) revealed the tetragonal rutile-type structure with a systematic average size reduction and unit cell volume tumescence, while enhancing the residual strain as the Eu-doped content increases. The surface defects that were caused by the incorporation of Eu ions within the surface oxide matrix were observed by high-resolution transmission electron microscope (HRTEM). The results of the response properties of sensors based on the different levels of Eu-doped SnO_2_ layered nanoarrays demonstrated that the 0.5 at % Eu-doped SnO_2_ layered nanorods arrays exhibited an excellent sensing response to methanal at 278 °C. The reasons of the enhanced sensing performance were discussed from the complicated defect surface structure, the large specific surface area, and the excellent catalytic properties of Eu dopant.

## 1. Introduction

In recent years, because of the aggravated atmospheric pollution, it was found that it has become more necessary to monitor the environmental gases. Therefore, an efficient, lightweight, and low-cost equipment system for detection and quantification of poisonous and hazardous gases has been progressively more important. Because of the easily mass-produced, good sensing properties, and lower cost, gas sensors based on semiconducting metal oxide, including SnO_2_ [1], ZnO [2], In_2_O_3_ [3], NiO [4], and so on, provide a very efficient means for monitoring volatile organic compound vapors (VOCs). Among of them, SnO_2_ has been extensively applied in gas sensors, ascribing to its higher chemical sensitivity, thermal stability, and lower cost [5]. However, several weaknesses, such as high working temperatures, poor selectivity, and limited maximum sensitivity are always lingering on it. Due to their excellent properties and novel applications in gas sensors, people have made great efforts to develop different nanostructured materials to improve the performances [6,7,8]. Especially, various novel nanostructures based on SnO_2_ semiconducting oxides, such as nano films [9], hollow nanofibers [10], nanocomposites [11,12], porous architecture [13], and so on, have been extensively investigated as a promising candidate for gas sensing materials.

One dimension (1D) oxide semiconductor nanocrystals are considered to be highly efficient materials for gas sensing due to the high mobility of conducting electrons, high surface to volume ratio, and sensitivity to surface chemical reactions in comparison to thin film gas sensors, which could lead to high gas response [14]. Since Law et al. reported that the photochemical sensor of individual SnO_2_ nanoribbons showed fast and sensitive response for detecting ppm-level NO_2_ at room temperature for the first time [15], the exploration of gas sensors focused on various 1D SnO_2_ nanostructures, such as nanowires [16], nanorods [17], nanobelts [18], nanotubes [19], and so on, have been attracting a great deal of interest for the detection of volatile organic compound vapors (VOCs). However, these products are usually difficult to effectively play its advantages for their random orientation. Because oriented geometry provides direct conduction paths for carriers to transport from the junction to the external electrode, nanoarray could be used as building blocks to fabricate functional devices [20]. Attributing to their ordered arrangement and inherent high surface-to-volume ratio, 1D array nanostructure is preferable to detect pollutant gases [21,22]. It could bring many surprising discoveries for us. Recently, a few prepared technologies of SnO_2_ nanoarrays, such as the hydrothermal method [23], thermal evaporation approach [24], chemical vapor deposition technology (CVD) [25], and so on, have already been reported. However, these strategies are performed on rigorous equipments, and multiple steps, which limits their application. Moreover, these methods always employ harsh, costly growth substrates, which hinder the fabrication of sensors and their sensing performance of the nanoarrays. Therefore, it is of great significance to developing new routes of synthesizing oriented SnO_2_ arrays without substrates. Among of them, the hydrothermal synthesis method is a promising candidate owing to their controllability, simplicity, and economy for large-scale preparation [26]. On the other hand, because of their superior electronic, optical, and magnetic properties, rare earth compounds have been widely used in high performance luminescent devices [27], varistor ceramics [28], catalysts [29], and so on. Doping with suitable elements of the rare earth compounds to modify the electronic, optical, and magnetic properties of metal oxide semiconductor materials is an effective strategy [30]. In recent years, many surveys have shown that the rare earth elements doped into the metal oxide structure can overcome disadvantages and improve the gas response, such as poor sensitivity and selectivity of sensors [31]. The effects of doping La [32], Yb [33], Pr [34], Ce [35], and Sm [36] have been reported, which indicate positive effect on enhancing gas sensing of SnO_2_ nanomaterials. Europium ion with 4f^7^ electronic configuration usually exists in the form of triply ionized ion (Eu^3+^) [37], which shows fast oxygen ion mobility and predominant catalytic properties. However, seldom research works reported the effect of the Eu dopant on the sensing performances of SnO_2_ nanomaterials. Furthermore, no attention has been yet focused on the layered Eu-doped SnO_2_ ordered nanorarrays that are synthesized by hydrothermal method and their gas sensitivity performances. Hence, it is necessary to explore the preparation and gas-sensing properties of layered Eu-doped SnO_2_ highly ordered nanorarrays for high performance sensor.

In the present work, undoped and different levels of Eu-doped layered SnO_2_ nanorods arrays were successfully synthesized under hydrothermal conditions without surfactants and substrates. The morphology, phase structure, microstructures, defect surface, and the residual strain of as-synthesized layered Eu-doped SnO_2_ ordered nanorarrays were systematically investigated. Then, the gas response experiments of Eu-doped SnO_2_ sensors towards methanal (The formula is labeled as HOCH) were carried out to envisage the effect of Eu dopant on the gas sensitivity performances when compared with the undoped SnO_2_ nanoarrays. By the end of the article, the possible reason for enhanced methanal response of the Eu-doped SnO_2_ nanorods arrays was also discussed.

## 2. Materials and Methods

### 2.1. Materials

Employed starting chemicals of sodium stannate four-hydrate (Na_2_SnO_3_·4H_2_O), europium chloride (EuCl_3_·6H_2_O), sodium hydroxide (NaOH), and absolute ethanol (C_2_H_5_OH) are analytical grade reagents that were purchased from the Sinopharm Chemical Reagent Co. (Shanghai, China), and without further purification. Deionized water was used throughout the experiments.

### 2.2. Syntheses of Eu-Doped SnO_2_ Layered Nanoarrays

Undoped and Eu-doped layered SnO_2_ ordered nanoarrays were synthesized via a route hydrothermal without any surfactants and substrates [38]. In brief, two kinds of 20 mL uniform solutions of 0.188 mmol Na_2_SnO_3_·4H_2_O and 7 mmol NaOH were firstly prepared, respectively, and then, the Na_2_SnO_3_·4H_2_O suspension was slowly added into the NaOH solution with ceaseless stirring. After that, 40 mL of absolute alcohol was gradually dipped into the mixed solution of the Na_2_SnO_3_ and NaOH with vigorous stirring for 60 min to form a semitransparent turbid solution, which shows a pH value of around 14 as measured by an extensive pH indicator paper. At room temperature, the prepared turbid solution was transferred to a 100 mL Teflon-lined stainless steel autoclave and then heated in an oven at 200 °C for 48 h. Following the next step, greyish white precipitate was collected and washed several times with deionized water and absolute alcohol, and dried in an oven at 80 °C for 24 h for further characterization. In order to obtain different doping levels of the Eu-doped SnO_2_ layered nanoarrays, a suitable amount of EuCl_3_·6H_2_O with the Eu to Sn atomic ratio of 0, 0.2, 0.5, 1, 1.5, and 3 at % was anteriorly added to the Na_2_SnO_3_·4H_2_O suspension solution, respectively.

### 2.3. Characterization of Eu-Doped SnO_2_ Layered Nanoarrays

The crystal phase and structure of Eu-doped SnO_2_ layered nanoarrays were identified by powder X-ray diffraction on a Rigaku D/MAX-3B (Rigaku Corporation, Tokyo, Japan) employing a copper target (*λ* = 1.54056 Å). In order to obtain further information from the XRD patterns, the peak shape was analysed using Rietveld refinement methods based on the Thompson-Cox-Hastings pseudo-Voigt function (TCH-pV) [39]: TCH-pV = *μL* − (1 − *μ*)*G*, (where *L* and *G* represent the Lorentzian and Gaussian peak functions, respectively, whereas *μ* is a mixing parameter). The deconvolution of the TCH-pV function provides the line widths of the Gaussian and the Lorentzian components given by FWHMG = (*U*tan 2*θ* + *V*tan *θ* + *W* + *Z*/cos 2*θ*)^1/2^ and FWHML = *X*tan *θ* + *Y*/cos *θ*, where *U*, *V*, *W*, *Z*, *X*, and *Y* are the refined parameters and can be used to determine the mean crystallite size (*D*) and the residual strain (*ε*) [40]. The line broadening of the instrumental contribution was corrected by the line width that was obtained from the refinement of a standard sample (SiO_2_ single crystal).

The morphology and composition of the as-prepared layered SnO_2_ nanoarrays were characterized by field emission scanning electron microscope (FESEM, Quanta 200, FEI Company, Hillsboro, OR, USA) attached with an energy dispersive spectroscopy (EDS) detector (Edax Octane super, Ametek company, San Diego, CA, USA). The microstructures and defect surface of the samples were observed by high-resolution transmission electron microscope high-resolution transmission electron microscope (HRTEM) employed on a transmission electron microscope (TEM, JEOL 2010, Akita City, Tokyo, Japan ). X-ray photoelectron spectroscopy (XPS) was recorded at room temperature in PHl X-tool using the Axis ultra spectrometer (Ulvac-Phi, Tokyo, Japan) at 10^−9^ Torr. During XPS analysis, Al-κ*α* X-ray radiation (1486.7 eV) was adopted as the excitation source and power was set to 250 W. Bonding energy was calibrated with reference to C1s peak (285.0 eV). The measured spectra were decomposed into Gaussian components with a Shirley background using the software XPSPEAK (Raymund W.M. Kwok, Hong Kong, China) by a least-square fitting method. A Micromeritics Gemini VII apparatus (Surface Area and Porosity System, Atlanta, GA, USA) was employed to obtain the nitrogen adsorption isotherm to determine the specific surface area of the products and the pore-size distribution obtained from the desorption branch of the isotherm using the corrected form of the Kelvin equation by means of the Barrett-Joyner-Halenda (BJH) method. Raman scattering spectra were obtained using a Renishaw inVIA Laser Micro-Raman spectrometer (Gloucestershire, UK). UV-Vis measurements were performed with a UV-2401PC spectrophotometer (Shimadzu, Japan). Thermoanalysis was performed on a simultaneous thermal analyzer of thermogravimetric analyzers-differential scanning calorimeters (TGA-DSC) (Q600, TA instrument, New Castle, DE, USA) heated from room temperature to 800 °C with a rate of 10 K/min.

### 2.4. Sensor Fabrication and Gas-Sensing Performance Tests

The gas sensor was chosen in the form of indirect-heating structure. As-prepared SnO_2_ Eu-doped nanorods arrays were used as a sensitive layer. Thick film of sensitive layer with a thickness of about 0.2–0.6 mm was coated on an alumina tube (2 mm in diameter and 4 mm in length) with Au electrodes and platinum wires. The detailed fabrication process of gas sensor was described as follows. Firstly, a proper amount of the as-synthesized Eu-doped SnO_2_ layered nanoarrays added several drops of terpilenol was slightly ground together in an agate mortar to form a homogeneous slurry. The resulting homogeneous slurry was coated on an Al_2_O_3_ tube by a small brush to form a thick sensing film between two parallel Au electrodes, and the embryonic device of sensor was obtained. After being dried in air, the embryonic device was annealed at 500 °C for 2 h, and a heater of Ni-Cr alloy coil with 0.1 mm in diameter was inserted into the tube to control the operating temperature from room temperature to 600 °C controlled by tuning heating voltage. Then, the electrodes and the Ni-Cr alloy wires were welded on six slender metal rods embedded in a plastic base. So far, the sensor had been fabricated. To improve the long-term stability, the fabricated sensor was kept at 500 °C for seven days in the atmosphere.

Gas sensing measurement system (JF02F, Jin Feng Electronics of Sino-Platinum Metals Co., Ltd., Kunming, China), which is controlled by a personal computer, was employed to test the gas-sensing properties. Its design is built on static gas test principle. The atmospheric air is invoked as the reference to dilute the target gas. The desired concentration was approximately obtained by the ratio of *V*_g_/*V*_c_ (*V*_g_ is the gas volume, and *V*_c_ is the tested chamber volume). The output resistance signal of the sensor was measured by a conventional circuit, in which the element was connected in series with an external resistor at a circuit voltage. Once the target gas was injected into the chamber, the sensor responded immediately with a resistance signal. The gas-sensing properties were assessed through the sensor response (S) that was defined as the ratio of *R*_a_/*R*_g_, where *R*_a_ denoted the resistance in reference air, while *R*_g_ denoted the resistance in target gases, such as methanal, acetone, ethanol, and gasoline. The range of the relative humidity of the tested atmosphere ranged from 40 to 70% at room temperature.

## 3. Results and Discussion

### 3.1. Characterization of Eu-Doped SnO_2_ Nanoarray

#### 3.1.1. TGA-DSC Thermoanalysis

**Figure 1:**
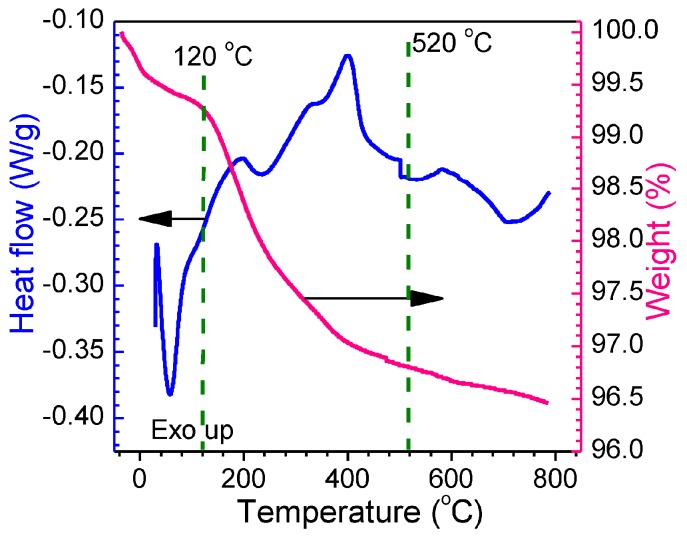
TGA-DSC thermography of 0.5 at % Eu-SnO_2_ nanoarrays.

Figure 1 shows the thermographic results of the as-synthesized 0.5 at % Eu-SnO_2_ nanoarrays product on simultaneous TGA-DSC thermoanalysis. A little weight loss process that was associated with the evaporation of absorbed water was observed and a larger endothermic peak was displayed on the DSC curve (blue curve) in the range of 20 to 200 °C. From 120 to 520 °C, it showed the rapid weight loss and a series of endothermic and exothermic peaks with the temperature increases, which were contributed to the complex physical and chemical reaction process, such as evaporation of trace organic impurities or hybrid products. After that, the horizontal straight line of weight loss indicated that no weight loss is too heavy. In the whole procedure, only 3.5% of the total weight was lost, indicating the stability of Eu-doped SnO_2_ nanoarrays products.

#### 3.1.2. SEM and EDS

**Figure 2:**
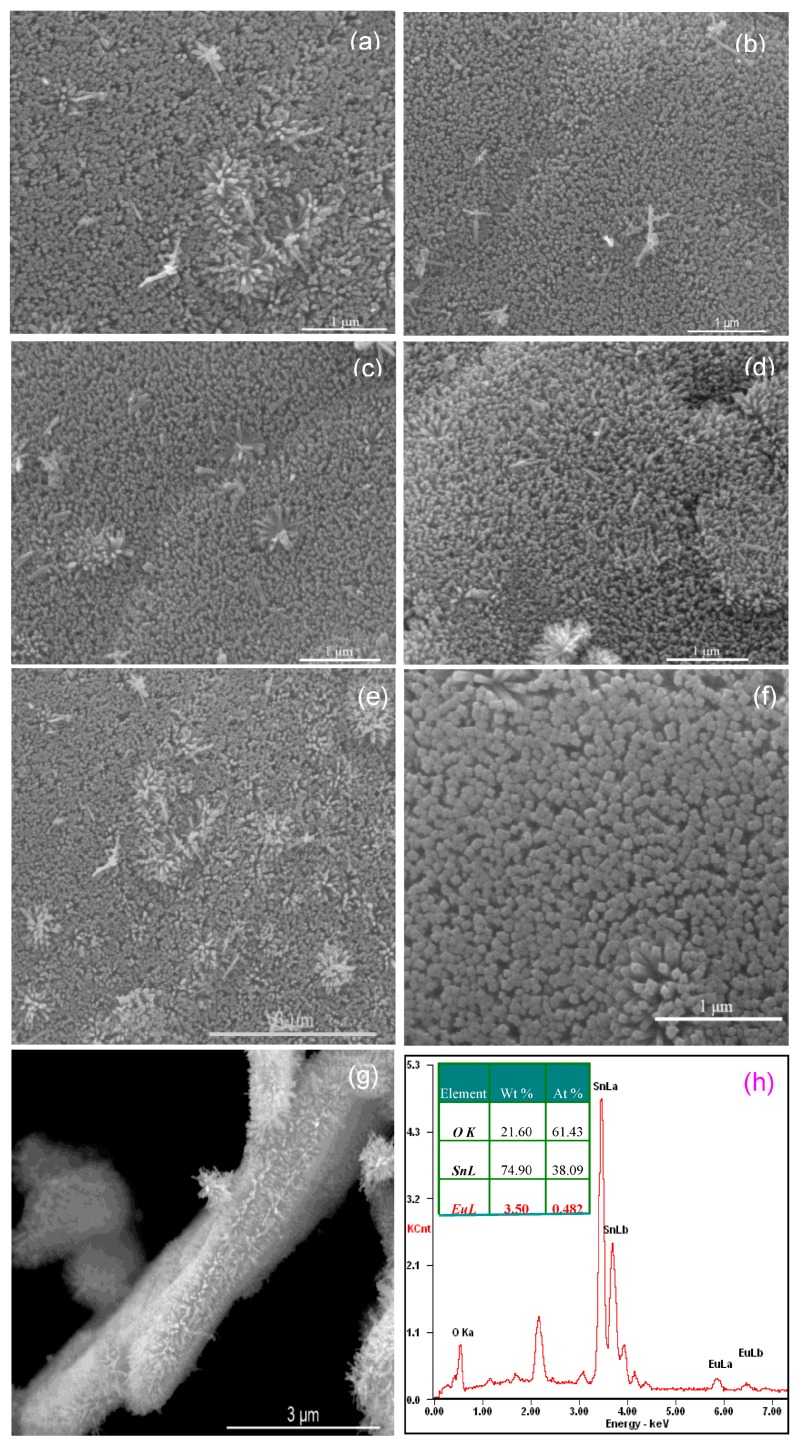
(**a**–**e**) Top-view SEM image of 0.2, 0.5, 1.0, 1.5, and 3.0 at % Eu-doped SnO_2_ nanorods array; (**f**) Undoped SnO_2_ nanorods array SEM image; (**g**) Cross-sectional SEM image of 0.5 at % Eu-doped SnO_2_ nanorods array; (**h**) EDX spectrum of 0.5 at % Eu-doped SnO_2_ nanorods array.

The morphologies of all as-prepared layered Eu-doped SnO_2_ nanoarrays were firstly characterized by SEM, and the results are shown in Figure 2. Top-view images display the large-area continuous, highly ordered dense SnO_2_ nanorods arrays. These fine round nanorods with narrow size distribution of all the samples of Eu-doped SnO_2_ layered nanorods arrays showed vertical and dense alignment in the array. It was statistically counted to be ca. 272, 318, 292, 279, and 267 μm^−2^ of the nanorods density in the plane of the 0.2, 0.5, 1, 1.5 and 3 at % Eu-doped SnO_2_ nanorods arrays (Figure 2a–e), and the diameter was approximately estimated to be 14, 9, 10, 12, and 17 nm, respectively. Contrastively, the nanorods density and the diameter of the undoped SnO_2_ nanorods array, as shown in Figure 2f, was statistically counted to be only ca. 203 μm^−2^ and was estimated approximately 25.8 nm, suggesting the undoped nanorods is thicker than that of the Eu-doped nanorods of SnO_2_ nanarrays under the same synthesized conditions. It indicates that the Eu dopant inhibits the growth of the SnO_2_ nanorods [37], resulting in slim SnO_2_ nanorods. A section edge of the 0.5 at % Eu-doped SnO_2_ layered nanoarray is displayed in Figure 2g. The slim nanorods exhibit a peculiar orderly arrayed structure of perpendicular and arranged uniform arrays. Furthermore, it was obvious that Eu-doped SnO_2_ nanoarray was tightly combined together by layered nanorods arrays. The nanorods on each array has a length of about 200–300 nm. These slim nanorods provide a large surface area to increase the surface trapping and heighten the surface reaction.

The composition of the Eu-doped layered SnO_2_ nanorods array was determined by the energy dispersive spectroscopy (EDX, Edax Octane super, Ametek Company, San Diego, CA, USA) spectrum. The EDS spectrum of the 0.5 at % Eu-doped layered SnO_2_ nanorods array is shown in Figure 2h. It demonstrates that the SnO_2_ sample is constituted by the elementary species of Sn, Eu, and O. The presence of gold (Au) and carbon (C) detected in the spectrum was introduced from the plating with gold and conducting glue. The quantitative atomic percentage of the compositional elements of oxygen (O), stannum (Sn), and europium (Eu) of 0.5 at % Eu-doped layered SnO_2_ nanorods array was presented as an inset of Figure 2h. The result of 0.482 at % Eu content in SnO_2_ nanoarray is within the experimental error range consisting with the initial ratio of experimental design.

#### 3.1.3. XRD

**Figure 3:**
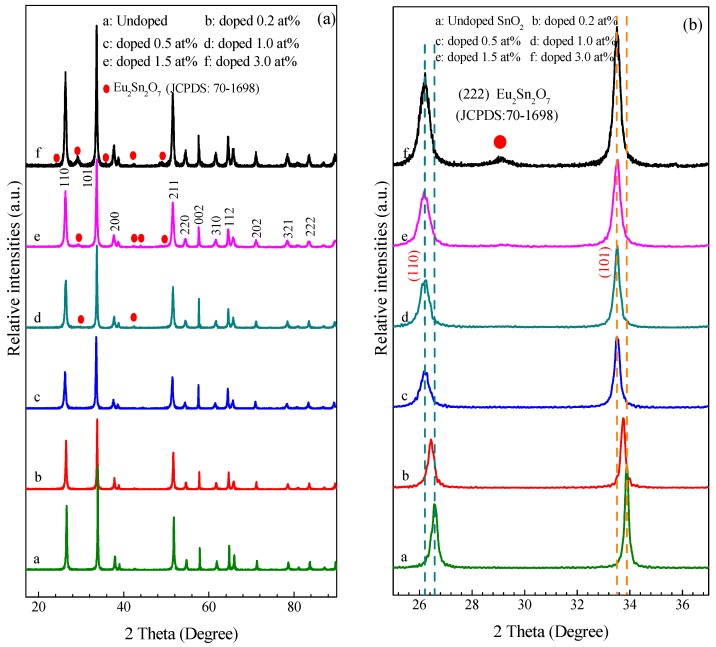
(**a**) X-ray diffraction (XRD) patterns of Eu-doped SnO_2_ nanorods arrays; (**b**) Enlarge XRD patterns showing the shift of (110) and (101) peaks.

X-ray powder diffraction (XRD) was employed and used to determine the crystal structure and phase of the products synthesized at different atomic ratios of Eu/Sn and corresponding XRD patterns are shown in Figure 3. For the undoped SnO_2_ nanoarrays sample, all of the diffraction peaks can be indexed to rutile-type SnO_2_ cassiterite structure with tetragonal lattice parameters *a* = *b* = 4.7386 Å, *c* = 3.1851 Å, consisting with the standard data file (JCPDS card No. 41-1445; space group, P4_2_/mnm(136)) [38]. As can be seen in Figure 3a, no evidence of any other crystalline or amorphous phase suggests the obtained SnO_2_ sample is good crystallinity and purity. Because no secondary phase was also found in the 0.2 and 0.5 at % Eu-doped SnO_2_ nanoarrays samples, it can be speculated that Eu-Sn-O solid solution formed in the SnO_2_. However, for the 1, 1.5, and 3 at % Eu-doped SnO_2_ nanoarrays samples, additional weak diffraction peaks positioned at 24.22°, 29.41°, 34.93°, 42.37°, 44.13°, or 49.17° can be observed in the XRD pattern curve of d, e, and f, and gradually become obvious with the increase of the Eu doping content. These diffraction peaks correspond to the crystalline phase of Eu_2_Sn_2_O_7_ (JCPDS card No. 13-0182) [37]. It indicates that the secondary crystalline phase of Eu_2_Sn_2_O_7_ had been formed in the SnO_2_ nanoarrays samples. This may be derived from the doped level of the 1 at % Eu dopant and above the doped level exceed the SnO_2_ saturation. The excess Eu atom reacted with the SnO_2_ to form the second phase of the Eu_2_Sn_2_O_7_. Paying attention to the relative intensities of all the layered SnO_2_ nanoarray samples, the higher intensity of (101) and (002) in comparison with the data provided by JCPDS file No. 41-1445 of SnO_2_ powders is observed [38]. The enhancement of the peak (002) intensity could ascribe to oriented growth in the [001] direction [38], indicating higher orientation degree of all the prepared SnO_2_ nanoarray samples.

Figure 3b depicts the enlarge XRD patterns of the corresponding to (110) and (101) plane of SnO_2_ array nanocrystals between 25° and 38° to penetrate the Eu effect on the characteristics of the diffraction peaks. An obvious variation of the intensity and full-width at half maximum (FWHM) with the Eu concentration ranged from 0 to 3% along (110) and (101) plane is observed. On the one hand, the peak position slightly shifted an angle Δ2*θ* (Δ2*θ* ~ 0.27°) towards lower side with an increase of the Eu content from 0 to 0.5 at % is observed, and then the peak position does not drift any more as the nominal Eu-content increaseing up to 3 at %. The shift of the peak suggests the increase of the lattice parameter caused by the tensile/compressive stress on the lattice derived from the Eu incorporation. It could be inferred that Eu^3+^ ions react with SnO_2_ to form a stable solid solution and the Eu^3+^ ions occupy the regular lattice site in SnO_2_ [37]. On the other hand, we can find that the peak intensity decrease and the line width of the XRD peaks increase as the nominal Eu-content increases to 0.5 at %, betokening that the crystalline size decreases gradually with the Eu-content increase. With the Eu-content increasing to 1.0 at %, the intensity of the peaks increase and the line width of the XRD peaks tends to decrease again. These findings could be attributed to the crystallite size reduction and/or the residual strain extent. It is well known that the final linewidth (*H*) is related to both effects (the crystallite size (*D*) and residual strain (*ε*)). Both of the parameters can be evaluated using the Williamson–Hall relation as follows [41]:

(1) $$H\cdot \cos\;\theta =\frac{k\lambda }{D}+4\varepsilon \cdot \sin\;\theta$$

where *λ* is the X-ray wavelength, *H* denotes the full width at half maximum, *θ* is the Bragg’s angle, *D* denoted the mean crystalline size, *ε* is the residual strain, and *k* is the dimensionless factor. The mean crystallite size and the residual strain is assessed from the linear regression of *H*·cos *θ* versus sin *θ* curve. Whereas, valid for a tetragonal structure, the structural parameters of the lattice constants have been calculated by using the following relations:

(2) $$\frac{1}{d^{2}}=\frac{h^{2}+k^{2}}{a^{2}}+\frac{l^{2}}{c^{2}}$$

where *d* is the interplaner distances, *h*, *k*, *l* are the Miller indices and *a* and *c* are the lattice constants. The list of the structural parameters obtained from the Rietveld refinements is collected in Table 1. The result shows the expected decrease of the mean crystallite size (*D*) as the nominal Eu-content increases from 0 to 0.5 at %, suggesting that the grain’s shrinkage due to the Eu dopant leads to supersaturation [42]. Similar behaviour has been observed in other literatures regarding the RE-doping of SnO_2_ nanoparticles [43]. The grain’s shrinkage is induced by the surface segregation (surface excess) of the dopant ions in the particle surface, which can be stabilized by the progressive decrease of the surface energy and can provide a barrier for the crystal growth diffusion [44]. Since the ionic radius of Eu^3+^ (0.0947 nm) is larger than the ionic radius of Sn^4+^ (0.069 nm), once the incorporating Eu^3+^ ions occupy the regular lattice SnO_2_ matrix could lead to distortion and stress in the cell and the lattice. As a consequence, the grains would be broken to cause the crystallite size decrease with respect to the Eu addition. However, the crystalline size increases slightly with the addition of the nominal Eu dopant above 1 at % level. The slight increase of the crystallite size could be ascribed to the formation of the Eu_2_Sn_2_O_7_ in the Eu-doped SnO_2_. The similar result of La_2_Sn_2_O_7_ guide SnO_2_ crystalline size increase in the La-doped SnO_2_ system had been reported by Weber [45]. Obviously, the Eu_2_Sn_2_O_7_ could induce the crystalline size increase and deteriorate the electric transport of SnO_2_ nanoarray to cause a negative effect on the gas-sensing properties of SnO_2_ nanoarrays.

It is clear from the values given in Table 1 that the lattice constants expand with the increase of the Eu-doped level, when compared with the undoped additional diffraction lines in the XRD pattern curve, as shown in Figure 3b. This result indicates that the substitution of Sn^4+^ ions by Eu^3+^ ions causes an increase in the lattice constants due to larger ions (Eu^3+^ = 0.947 Å) replacing smaller ions (Sn^4+^ = 0.69 Å). It is consistent with the result of theoretical calculation as to the strong preference for the substitutional solution of trivalent, rare-earth dopants in SnO_2_, and the creation of oxygen vacancies to achieve charge neutrality [46]. Therefore, the expanded lattice constants strongly suggest the formation of Eu-SnO_2_ solid solution. Whereas, the opposite behaviour of the *c*/*a* ratio showing an increasing tendency as the Eu content increases, while the internal parameter of the rutile structure (*u*) decrease, which suggests an interesting structural change of the intrinsically flattened octahedron of oxygen atoms surrounding the Sn atoms [42]. Meanwhile, the almost linear increase of the *c*/*a* ratio with the Eu level increases, indicating that an anisotropic expansion of the unit cell along the c axis, is favoured by the Eu doping. As a result, the unit cell volume becomes larger as the nominal Eu-content increases, as presented in Table 1. The same tendency is observed for the residual strain, originated in the distortions in the crystal lattice because the ionic radius of Eu^3+^ is larger than the ionic radius of Sn^4+^. The incorporation of the Eu^3+^ ions into the matrix could lead to larger mismatch. Therefore, the residual strain (*ε*) must be related to oxygen vacancies and site disorder, which are conducive to gas adsorption and enhanced gas response.

#### 3.1.4. TEM and HRTEM

**Figure 4:**
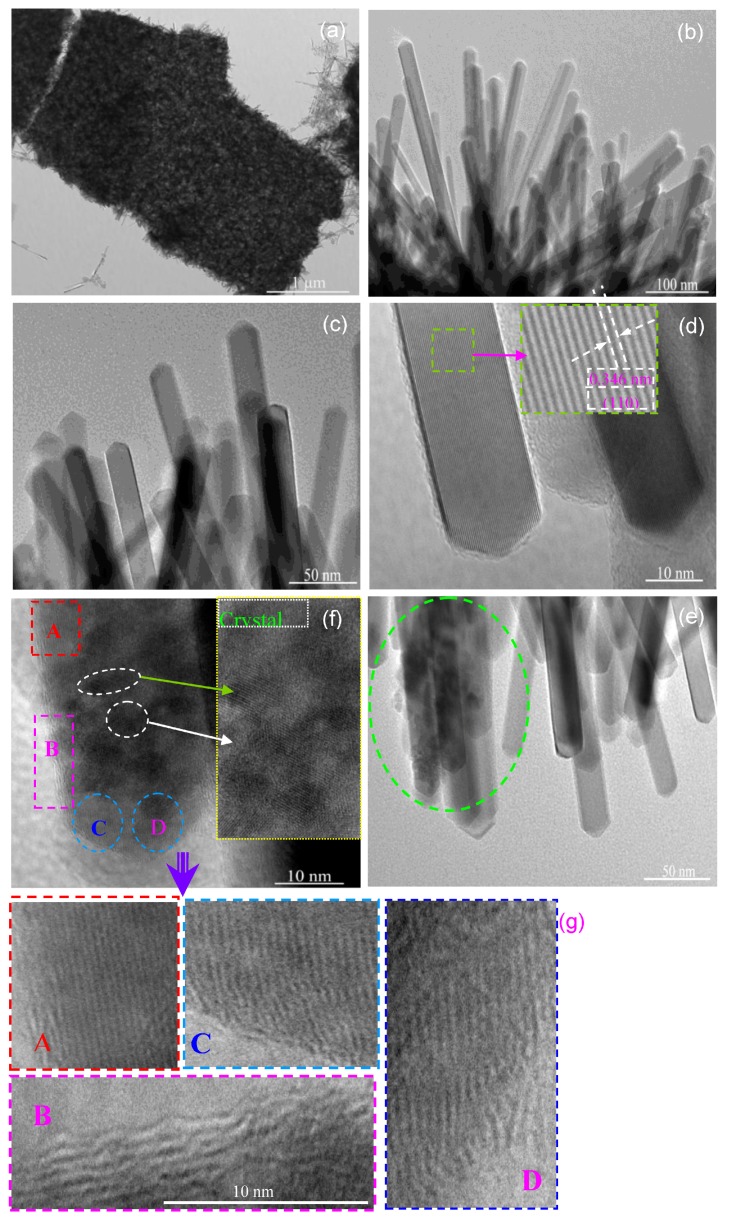
TEM images of 0.5 at % Eu-doped SnO_2_ nanorod array: (**a**) Low magnification TEM image of a section nanorods arrays; (**b**) Top TEM image of nanorods arrays; (**c**) High magnification TEM image of individual nanorods; (**d**) HRTEM image of an individual Eu-doped SnO_2_ nanorod. Inset: an enlarge lattice fringes; (**e**) TEM image of defective nanorods; (**f**) HRTEM image of an individual defective SnO_2_ nanorod adhered by crystal grains and marked as A, B, C, and D area, respectively. Inset: an enlarge lattice fringes of crystal grains; (**g**) Enlarge HRTEM image of the marked area A, B, C, and D showing twists and turns, distorted, and discohered lattice fringes, accordingly.

In order to further obtain the microstructures of the Eu-doped SnO_2_ nanoarrays samples in detail, TEM was carried out and the result was shown in Figure 4. Figure 4a shows a typical low-magnification TEM image of the 0.5 at % Eu-doped layered SnO_2_ nanoarrays, where the SnO_2_ nanoarrays assembled by nanorods can be clearly observed. The SnO_2_ nanorods stood on the border of the arrays, as shown in Figure 4b. Rods-like nanostructure of the prepared SnO_2_ nanorods array can be clearly seen in Figure 4b. An enlarged peaked top TEM image of SnO_2_ nanorods array is shown in Figure 4c, confirming that the Eu-doped SnO_2_ nanoarrays consists of rod-like solid with a smooth surface and a conical top. The straight nanorods have a diameter of around 11 nm. Figure 4d shows an high-resolution transmission electron microscope (HRTEM) image of high quality single SnO_2_ nanorod and clear lattice fringes parallel to the length of the nanorod. Corresponding to the (110) lattice planes of SnO_2_ in tetragonal cassiterite structure, the survey of 0.343 nm space of adjacent lattice fringes from the inset shown in Figure 4d is significantly larger than the classical value of 0.335 nm of bulk SnO_2_, which is attributed to the expansion of lattice parameter. These findings further suggest the tensile/compressive stress in the microstructure and the exposed facet (110) of the SnO_2_ nanorods oriented growth preferentially along the [001] direction [47].

It is valuable that some defective nanorods that are shown in Figure 4e are obviously observed. Figure 4f shows a HRTEM image of a single defective SnO_2_ nanorod. The clear lattice fringes illustrate the single SnO_2_ nanorod is of a high quality rutile crystal. As shown in the upper-right inset of Figure 4f, some crystal grains estimated to be of 5 nm adhered to the surface of the nanorods were observed. Although the microstructures of these crystal grains need to be further observed in detail, it is reasonable to suspect that it is the phase of Eu_2_Sn_2_O_7_ crystalline, since the diffraction peaks of Eu_2_Sn_2_O_7_ phase were detected in the XRD measurement, as shown in Figure 3. Focus on the lattice fringes of the SnO_2_ defective nanorod, it is palpable that the overall structure of SnO_2_ nanorod is not uniform. For convenient description, they are marked as A, B, C, and D area, as shown in Figure 4f, and the enlarged HRTEM images are shown in Figure 4g correspondingly. The enlarge HRTEM image of the marked area A depicts a vague uniform structure (comparison with the Figure 4d). The space of 0.349 nm between two adjacent lattice fringes, corresponding to the exposed facet (110) lattice planes of SnO_2_ in tetragonal cassiterite structure, is obviously larger than the usual value of 0.335 nm of a bulk SnO_2_. The enlarged HRTEM image of the SnO_2_ nanorod edge (the marked area B) and the top area (the marked as C and D), as shown in the Figure 4g, depicts the lattice fringes and reveals approximate 0.354 and 0.352 nm space between two adjacent lattice fringes, which also could correspond to the (110) lattice planes of the tetragonal cassiterite structure SnO_2_. However, it is obvious that the edge lattice fringes of the SnO_2_ nanorods are twists and turns, while the lattice fringes of the top area show distorted and discohered, which evidence dislocations and extended defects inside of the SnO_2_ nanorod after doping Eu. The entry of the Eu-ion originating distortions in the crystal lattice evidenced by the increase of the lattice strain (*ε*) leads to mismatch. Due to the larger ionic radius of Eu^3+^, incorporating Eu^3+^ ions into the matrix most likely leads to some distortion and stress in the cell and the lattice, which is supported by the XRD results, as shown in Figure 3b. As a result, the lattice fringes of SnO_2_ nanorod might be distorted and discohered in the crystallite with respect to the Eu dopant.

The formation mechanism of the highly ordered SnO_2_ layered nanorods arrays had been discussed in our previous work [38]. It is elucidated that SnO_2_ nanorodarrays employed the Ostwald ripening process, followed by a mechanism named as in situ oriented growth. The formation and growth of nanoarray strongly depended on the experimental conditions including pH value, concentration, growth temperature, reaction time, and so on. The results of the SEM and XRD in the present work showed that the dopant Eu does not only affect the formation of the layered SnO_2_ nanorods array, but also it will restrain the nanorods grew up and lead the lattice to mismatch. The possible procedure may be proposed as the following:

(3) $$\left\{ \begin{array}{l} {\mathrm{Sn}}^{4+}+{\left(\mathrm{OH}\right)}^{-}\;\to {\mathrm{Sn}(\mathrm{OH})}_{4}\\ {\mathrm{Eu}}^{3+}+{\left(\mathrm{OH}\right)}^{-}\to {\mathrm{Eu}(\mathrm{OH})}_{3} \end{array}\right.\Rightarrow {\left(\mathrm{Eu})\mathrm{Sn}(\mathrm{OH}\right)}_{4}\overset{\mathrm{Nucleation}\;\mathrm{growth}}{\to }{(\mathrm{Eu})\mathrm{SnO}}_{2}↓+2H_{2}O$$

#### 3.1.5. UV-Vis Spectrum

**Figure 5:**
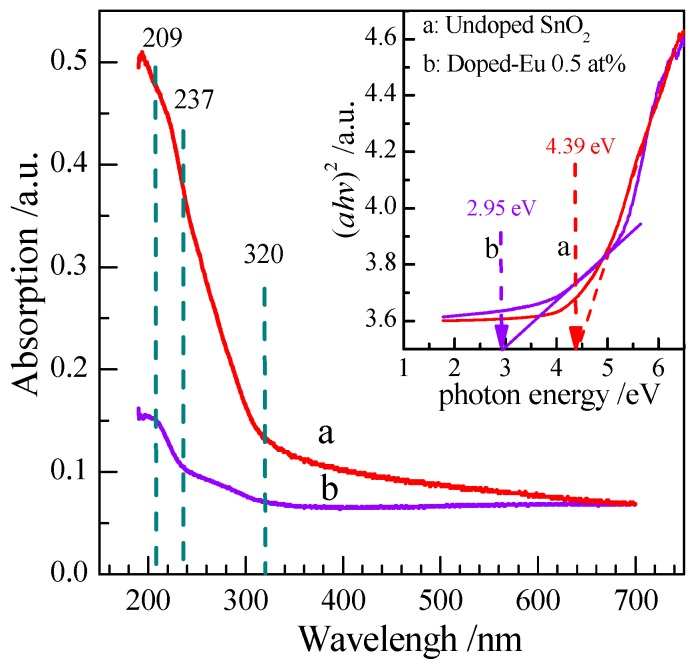
UV-vis spectra of undoped and 0.5 at % Eu-doped SnO_2_ nanorods array.Inset: (*αhν*)^2^ vs. photon energy plots.

The UV-vis spectra of the undoped and 0.5 at % Eu-doped SnO_2_ nanorods array samples are shown in Figure 5. High-energy shift of an absorption edge could be estimated for the nanorods array. The nonlinear characteristics of the plots suggest the direct transition nature of SnO_2_ materials. The feature of the absorption edge located at around 300 nm confirms the SnO_2_ structure of ordered nanoarray. A strong absorption of the pristine SnO_2_ nanoarray appeared near the visible-light region in the vicinity of 237 nm, while spectrum of the 0.5 at % Eu-doped SnO_2_ sample appears a broad and weak absorption region in the wavelength range of 209–320 nm. The optical band gap (*E*_g_) can be determined by using the Tauc and Menth’s law based on the optical absorption spectra [48]. The absorption coefficient obeys the following energy dependence:

(*αhν*)*^n^* = *B*(*hν* − *E*_g_) (4)

where *hν* denotes the photonic energy, *α* is the absorption coefficient, *n* is equal to 2 for SnO_2_, *E*_g_ denotes the band-gap energy, while *B* is a constant related to the material. The plot of (*αhν*)^2^ vs. *hν* based on the relation (4) is presented in inset of the Figure 5. The corresponding value of the optical band gap (*E*_g_) for bulk SnO_2_ was 3.65 eV, while 4.39 and 2.95 eV for undoped and 0.5 at % Eu-doped SnO_2_ nanorod arrays, respectively. The increase in the band-gap of undoped SnO_2_ nanorod arrays in comparison with the bulk counterparts suggests blue-shifted, being ascribed to the quantum confinement effect of nanostructure [49]. The decrease in the band-gap is reflected by the red shift in the absorption spectra. The variation in band-gap due to Eu doping could be attributed to the Mosse–Burstein (MB) effect and the appearance of the Eu–Sn metallic compounds [50]. The narrowing band-gap can prompt an electronic transport rate, which is conducive to the gas adsorption/desorption. It motivates us to explore the Eu-doped SnO_2_ nanorods arrays for gas sensing.

#### 3.1.6. X-ray Photoelectron Spectroscopy (XPS)

**Figure 6:**
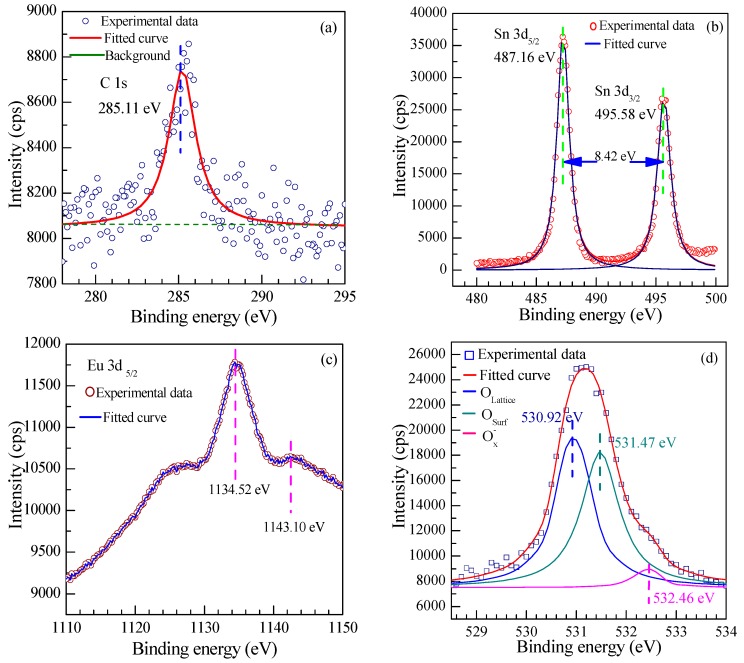
The High-resolution X-ray photoelectron spectroscopy (XPS) survey spectra of 0.5 at % Eu-doped SnO_2_ nanorods array: (**a**) C1s; (**b**) Sn3d; (**c**) O 1s; and, (**d**) Eu 3d region.

The surface chemical bond configuration and the composition of the Eu-doped SnO_2_ nanoarrays were determined by XPS spectra. The high-resolution XPS spectra of the 0.5 at % Eu-doped SnO_2_ nanoarrays are shown in Figure 6. The binding energy of the C1s at 285.11 eV caused by the contamination of the instrument is shown in Figure 6a. Figure 6b displays the XPS spectra survey of Sn 3d that the Sn 3d_5/2_ peaks occur at 487.32 eV, and the Sn 3d_3/2_ peak is located at 495.78 eV, respectively. It demonstrates that the energy difference between Sn 3d_5/2_ and Sn 3d_3/2_ peaks is 8.42 eV, being in good agreement with the energy splitting reported for bulk SnO_2_ [51]. The Eu 3d XPS spectra of the 0.5 at % Eu-doped SnO_2_ nanoarrays presented in Figure 6c exhibits an intense peak of the Eu 3d_5/2_ located at 1134.52 eV along with a shake-up weaker peak at 1143.10 eV. This peak is assigned to the Eu^3+^ state, as is reported in earlier XPS studies of trivalent compounds of Eu [37].

From the O 1s spectrum of 0.5 at % Eu-doped SnO_2_ nanoarrays as shown in Figure 6d, it is obvious that the O(1s) feature is wide and asymmetric due to various coordinations of oxygen on the surface of the nanorods. It can be deconvoluted into three well-defined Gaussian-like peaks, located at 530.92, 531.47, and 532.46 eV, respectively, which reveal the presence of three types of oxygen-related species. The first component of 530.92 eV should correspond to the crystal lattice (O_lattice_) with chemical states Sn^4+^ (SnO_2_) under completely oxidized stoichiometric condition. Although, the origin of the second component located at around 531.47 eV is controversial, it is accepted that it can be assigned to O_Surf_^2−^ ion of the crystal lattice in the SnO_2_ surface, ascribing it to the oxygen vacancy (V_O_), oxygen interstitial (O_i_) and oxygen antisite (O_Sn_) in the oxygen deficient regions [52]. Though the O_Surf_^2−^ is not reactable with the tested gas, it can increase the holes concentration to be attributed to absorb the oxygen from the atmosphere. The highest binding energy of oxygen species located at 532.29 eV, which is usually not easy to observe directly, corresponds to chemisorbed and dissociated oxygen species (O^−^ and O^2−^) [53], evidencing the rich chemisorbed oxygen species (O^−^ and O^2−^) on the surface of SnO_2_ nanoarrays after doping the Eu. Since the chemisorbed oxygen species (O^−^ and O^2−^) are reactable with the gas and then enhance the holes concentration [17], rich adsorbed oxygen ions would contribute to the gas response.

#### 3.1.7. Brunauer-Emmett-Teller (BET)

**Figure 7:**
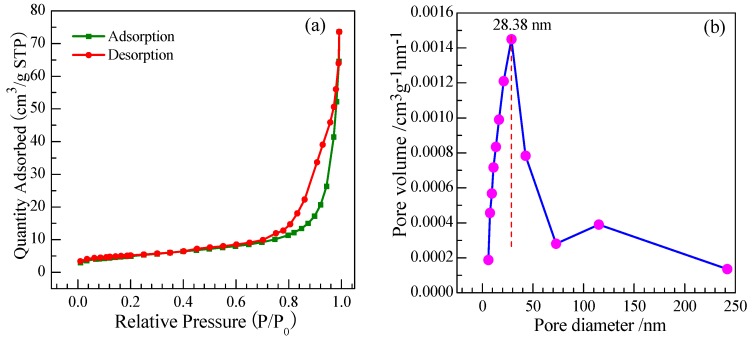
(**a**) N_2_ adsorption-desorption isotherm; (**b**) Barrett-Joyner-Halenda (BJH) pore size distribution plot.

To further investigate the pore size distribution as well as the surface area of the as-synthesized SnO_2_ nanoarrays product to predict the performances of gas response, the Brunauer-Emmett-Teller (BET) measurement was performed. Figure 7 shows the representative N_2_ adsorption and desorption isotherm and the corresponding BJH pore size distribution plot of the 0.5 at % Eu-doped SnO_2_ nanorods array. As shown in Figure 7a, the isotherm exhibits a hysteresis loop that is associated with the filling and emptying of the 0.5 at % Eu-doped SnO_2_ nanorods arrays by capillary condensation. It suggests that the SnO_2_ nanorarray exists a larger pore volume between the nanorods. The pore size distribution curve of the 0.5 at % Eu-doped SnO_2_ nanorarray, as shown in Figure 7b, demonstrates a broad peak ranging from 5 nm for up to 240 nm with a maximum at 28.38 nm. It suggeststhat the SnO_2_ nanorarray is assembled by thinner nanorods and has a capacious pore space among the nanorods, indicating a large effective surface area of nanoarrays. Actually, the specific surface area of the 0.5 at % Eu-doped SnO_2_ nanorarray was calculated to be 27.95 m^2^·g^−1^ by BET method. Therefore, it is expected that the Eu-doped SnO_2_ nanorarray with a large number of mass transport channels and a large surface area could improve the performance of gas response.

### 3.2. Methanal-Sensing Performance of Eu-Doped SnO_2_ Nanorarray

Due to the ordered structure of the array arrangement, the achieved Eu-doped SnO_2_ nanorarray here would be in favour of improving gas-sensing performances. To verify this view, we took the undoped and Eu-doped SnO_2_ nanoarray products as sensing materials, the gas-sensing performances of the Eu-doped SnO_2_ nanoarray were systemically tested.

#### 3.2.1. Relationship between Response and Operating Temperature

**Figure 8:**
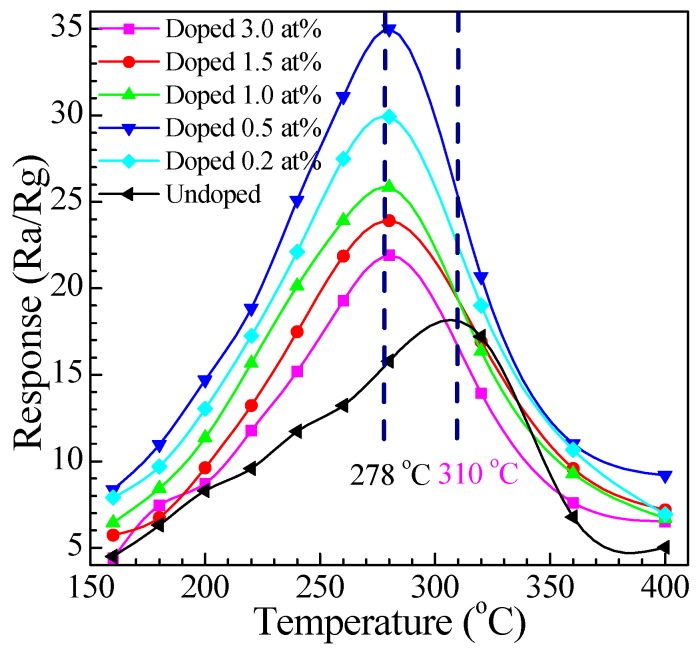
Responses of Eu-doped SnO_2_ nanorods array sensors toward 200 ppm methanal at different operating temperature.

It is well known, the operating temperature and the dopant level strongly affect the gas response. In order to observe the effects of Eu dopant on the response for methanal, we systematically performed experiments of SnO_2_ nanoarray with doping various Eu contents exposed to tested gas at different operating temperatures. Figure 8 exhibits the relationship between the responses of all sensors to 200 ppm methanal gas at different operating temperature. From the curve, all of the sensors show the same changing trend: the response rapidly increased, and reached a maximum at a certain temperature, and then rapidly decreased with the increase of the temperature. This physical phenomenon is attributed to the adsorption of oxygen ions on the SnO_2_ surface. As an *n*-type metal-oxide semiconductor, SnO_2_ can adsorb oxygen from the atmosphere in the O_2_^−^, O^−^, or O^2−^, which depend on the temperature. The adsorbed O^−^ is the most interesting for sensor due to it is more reactive, and thus makes the material more sensitive to the reducing gas. The surface preferentially adsorbs O^2−^ at a relative lower temperature and the sensitivity is consequently very small due to the slower reaction activity. With the increase of the temperature, O^−^ adsorption gradually becomes the dominant process, which can heighten the reaction activity to increase the gas response. However, if the temperature increases too much, progressive adsorption of all the oxygen ionic species previously adsorbed occurs and the response decreases [54]. It can be clearly seen from Figure 8, all of the Eu-doped SnO_2_ nanoarrays sensors demonstrated a considerably improved response in comparison to the undoped SnO_2_ nanoarrays sensor. The optimum responses obtained at about 278 °C for 0.2, 0.5, 1, 1.5, and 3.0 at % Eu-doped SnO_2_ nanoarrays sensors are 30, 35, 25, 23, and 21, respectively. However, the maximal response of the undoped SnO_2_ nanoarrays to 200 ppm methanal was only about 18 at 310 °C. This result indicates that the optimal working temperature of all the Eu-doped SnO_2_ nanoarrays sensors are almost 278 °C, which descends about 22 °C in contrast to the undoped sensor. It could be explained by the activation energy decrease due to energy barrier height and excellent electrocatalytic effect of Eu dopant, resulting in depression of the optimum temperature for the gas sensing response.

On the other hand, Figure 8 also indicates that the Eu dopant can improve the response toward methanal. An appropriate level of Eu dopant can greatly improve the gas sensing properties of layered SnO_2_ nanoarray. As shown in Figure 8, the sensor based on 0.5 at % Eu–doped SnO_2_ nanoarrays exhibited the highest response to methanal and the response amplitude run up to 35 at 278 °C, while the response of the 0, 0.2, 1.0, 1.5, 3.0 at % Eu–doped SnO_2_ nanoarrays sensors are about 18, 30, 25, 23, and 21, respectively. The response of the 0.5 at % Eu–doped SnO_2_ nanoarray is more than two times than that of the undoped SnO_2_ nanoarray. Therefore, this result indicates that the level of 0.5 at % Eu-doped is an optimal doping level in our report.

#### 3.2.2. Dynamic Response to Different Concentration

**Figure 9:**
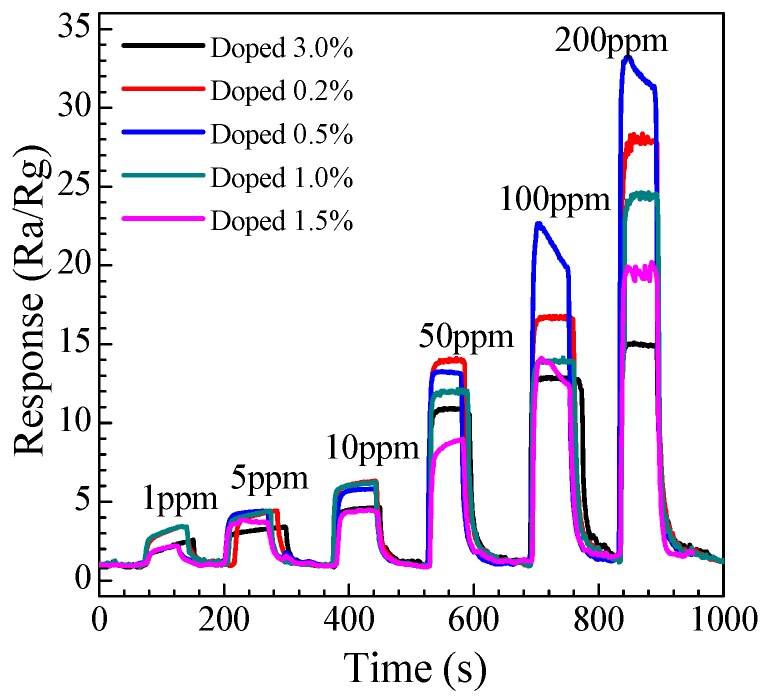
Typical dynamic response and recovery characteristic curves of the Eu-doped SnO_2_ nanorods array sensors to methanal ranging from 1 to 200 ppm at 278 °C.

The gas dynamic response of the Eu-doped SnO_2_ nanorods arrays gas sensor was measured. The representative gas dynamic response of the Eu-doped SnO_2_ nanorods arrays to methanal with the concentration range of 1–200 ppm at 278 °C and the result is shown in Figure 9. Corresponding to methanal concentrations of 1, 5, 10, 20, 50, 100, and 200 ppm, seven cycles recorded successively demonstrates that the response amplitude gradationally increases with the gas concentration increase. As can be seen from Figure 9, not only a higher response, but also good repeatability is observed. Figure 9 reveals the required dynamic response of the sensor in the presence of the methanal gas. Moreover, as shown in Figure 9, the sensor presents a considerable response of 3.8 to 1 ppm methanal at the optimum temperature of 278 °C, suggesting the as-prepared SnO_2_ nanoarray product is favourable to detect methanal with low concentration. The results are repeatable and reproducible, making the Eu-doped SnO_2_ nanorods arrays sensor to be the potential candidate for practical detector for methanal.

#### 3.2.3. Gas-Sensing Response and Recovery Time

**Figure 10:**
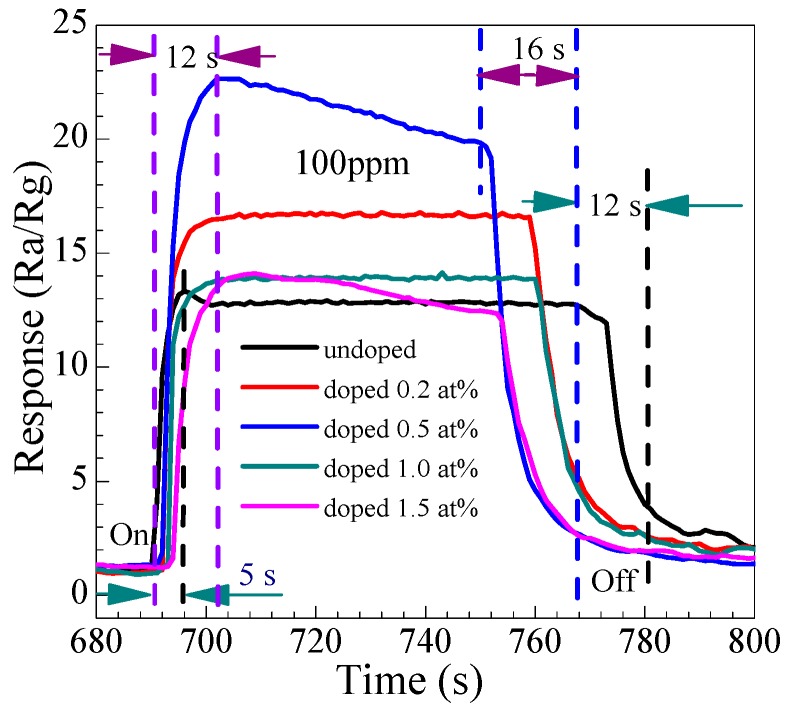
The response and recovery transient time examined of the Eu-doped SnO_2_ nanorods arrays sensors.

For evaluation of the gas performance, the gas-sensing response and recovery time (i.e., the time required to achieve 90% of the stable response) is an important consideration. The response times (*t*_on_) and recovery times (*t*_off_) were calculated from the response–time data shown in Figure 10, which is the transient response of the sensors to 100 ppm methanal at 278 °C. Obviously, the response time is similar for different Eu-doped nanoarray SnO_2_ sensors. Based on Figure 9, the sensing response and recovery time of the Eu-doped undoped SnO_2_ nanoarrays sensor is determined to be about 12 and 16 s, respectively. However, the response and recovery time of the undoped SnO_2_ nanoarrays sensor is only about 5 and 12 s, respectively. It suggests that both the response and recovery become slower after doped Eu, which could be attributed to the decrease of the chemical reaction rate due to the Eu dopant elevate the activation energy of chemical reaction, resulting in prolonging the response and recovery time. Similar results have been reported by G.T. Ang [55] and S.L. Shi [56].

#### 3.2.4. Relationship between Responses and Concentrations

**Figure 11:**
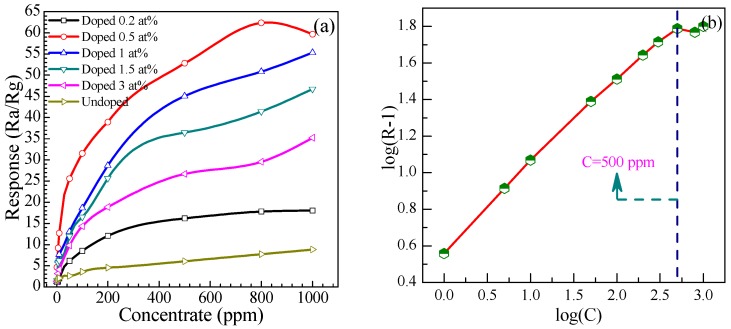
(**a**) Gas response to methanal concentrations; (**b**) The linear relationship between *R* and *C* in logarithmic forms of 0.5 at % Eu-doped SnO_2_ nanorods array sensor.

The responses of these sensors fabricated using the undoped and Eu-doped SnO_2_ nanoarrays at methanal concentrations from 1 to 1000 ppm were calculated at 278 °C in Figure 11. As depicted, the responses of all the sensors increase with the gas concentrations. At the beginning of 500 ppm, the response rapidly increases. Above 500 ppm, the response still increases, however, less rapidly. It indicates that the responses become more or less saturated. Particularly, sensor based on 0.5 at % Eu-doped SnO_2_ nanoarray exhibit a much higher response than undoped and other Eu-doped SnO_2_ nanoarrays. It is noteworthy that the curves of the response versus concentration are not linear, indicating that our measurements include a wide concentration range. The response law of metal-oxide gas-sensitive resistor is empirically expressed as: *R* = *a*[*C*]*^b^* + 1 [57], where [*C*] denotes the target gas concentration, *a* is a experimental parameter, and *b* is the surface species charge parameter with a value of 1 or 0.5 as determined by the chemisorbed oxygen species (O^−^ and O^2−^). Based on the above formula, we can derive the following equation log(*R* − 1) = *b*log(*C*) + log*a*. It exhibits the linear relationship between *R* and *C* in logarithmic forms. As shown in Figure 11b, in log–log plot according to the responses versus concentration curves of the 0.5 at % Eu-doped nanoarray showed the linear relationship in the concentration ranged from 1 ppm to 500 ppm. Moreover, the value of *b* is 0.91 for 0.5 at % Eu-doped nanoarray obtained by least squares fitting. It demonstrates that the sensitivity linearly increased as the methanal concentration increases with a R-square confidence value above 0.90. These results suggest the dominant adsorbed oxygen species is O^−^ ions.

#### 3.2.5. Selectivity of Sensor

**Figure 12:**
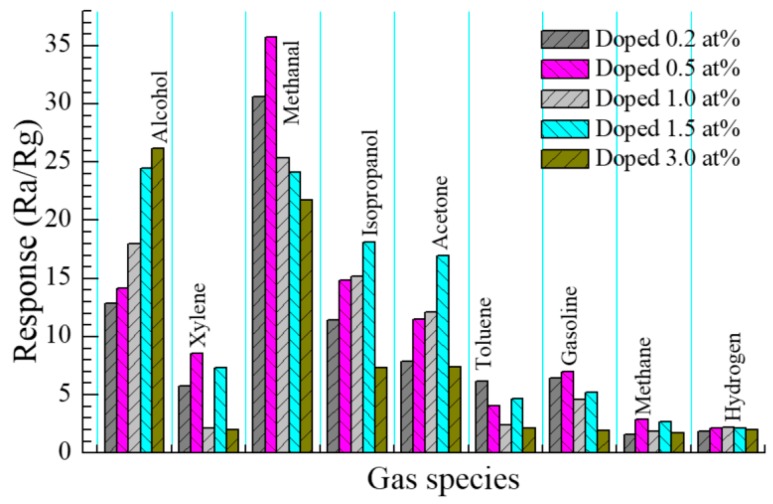
Response of gas sensor based on 0.5 at % Eu-doped SnO_2_ nanorods arrays to various gases (200 ppm) including alcohol, xylene, methanal, isopropanol, acetone, toluene, gasoline, methane, and hydrogen at 278 °C.

A selectivity test was performed at the optimal operating temperature of 278 °C to further inspect the sensing properties of the 0.5 at % Eu-doped SnO_2_ nanoarray. We performed experiments of the Eu-doped SnO_2_ nanoarrays with doping different contents that were exposed to 200 ppm various tested gases (alcohol, xylene, methanal, isopropanol, acetone, toluene, gasoline, methane, and hydrogen), and the results are shown in Figure 12. It is evident that the gas sensor of the 0.5 at % Eu-doped nanoarrays responses well to methanal when compared with the other gases, which confirms good selectivity.

### 3.3. Enhanced Gas Sensing Performance Mechanism

**Figure 13:**
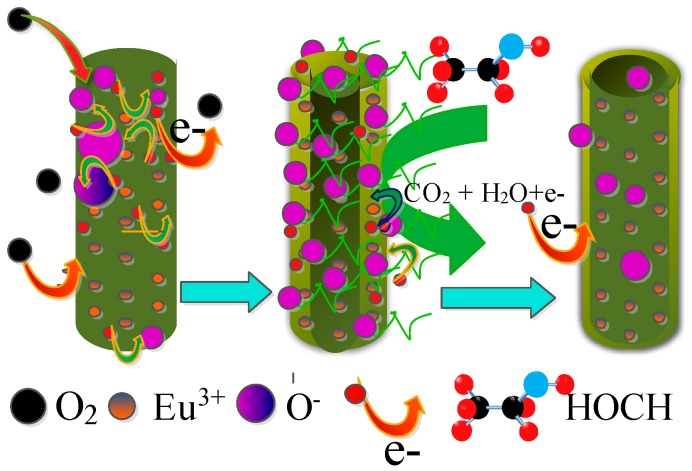
Schematic illustration of the gas sensing mechanism of the Eu-doped SnO_2_ nanoarry sensors.

As a surface controlled n-type metal-oxide semiconductor, the electrical conductivity of SnO_2_ strongly depends on the chemical reactions on the surface of tin dioxide [58]. At present, the gas sensitive mechanism based on surface chemical reactions, which lead to the change of resistance, is widely accepted. When the SnO_2_ nanorods arrays are exposed to the atmosphere, oxygen molecules are adsorbed on the surface of SnO_2_ and further evolve into oxygen species of O_2_^−^, O^−^, or O^2−^ by capture electrons from the conduction band as described by following Equation (5) [59]. Thus, it causes an ordered electron depletion layer on the surface of the SnO_2_ nanorods, resulting in the decrease of electron concentration and the increase of resistance. Upon exposure to target gas, such as methanal, the methanal molecules react with the adsorbed oxygen species, which will release the trapped electrons back to the SnO_2_ conduction band. It will lead to the contraction of the depletion layer and the decrease of the resistance. Once the gas is completely drained, the sensor will be exposed to the atmosphere again and refreshed by air. The reaction between methanal and ionic oxygen species can be depicted as in Equation (6) [60]. The Schematic illustration of the SnO_2_ nanorods array sensing mechanism is shown in Figure 13.

(5) $$O_{2(gas)}\to O_{2(\mathrm{ad}s)}+e\to O_{2(\mathrm{ads})}^{-}+2e\to 2O_{\left(\mathrm{ads}\right)}^{-}+2e\to 2O_{\left(\mathrm{ads}\right)}^{2-}$$

HOCH + O^−^/O_2_^−^/O^2−^ → CO_2_ + H_2_O + e^−^ (6)

The enhancement of methanal-sensing performance of highly ordered SnO_2_ nanorods layered array doped by Eu can be attributed to the following aspects.

Based on the above discussion of the gas sensitive mechanism, it indicates that a rich adsorption of oxygen species (O^2−^, O^−^, and O_2_^−^) is a critical factor for improving the response of the sensors because it can effectively promote the gas reactions on the SnO_2_ surface. Thus, it is obvious that a large specific surface area of SnO_2_ can effectively improve the gas response due to providing more active sites to improve the adsorption of the oxygen species. As confirmed that the capacious pore space and a large effective surface area of the nanorods arrays are significantly beneficial for their gas sensing performance [61], the ordered array layered structure exactly accounts for their better performance than the SnO_2_ nanorods as methanal sensor [21]. Especially, thinner nanorods and densely aligned SnO_2_ nanorods arrays can provide a large surface area to facilitate the adsorption of oxygen ions and gas molecules on the surface. From the results of the SEM and XRD, as an effective grain growth inhibitor, the doping Eu causes the crystallite size decrease and slender SnO_2_ nanorod arrays were obtained. Slender SnO_2_ nanorod arrays provide a higher specific surface area, which improves the adsorption of oxygen species. On the other hand, the replacement of the Sn^4+^ by the Eu^3+^ leads to the unmatched lattice matrix of the SnO_2_ surface. It could cause more surface defects, such disordered structures, puckers, and boundaries (as shown in Figure 4), which also can increase the specific surface area to afford more active sites for gas adsorption. Thus, the rich oxygen species of O^2−^, O^−^, and O_2_^−^ absorbed on the surface of the Eu-doped SnO_2_ nanoarrys is observed by the result of the XPS (as shown in Figure 6). Therefore, doping Eu is conducive to the oxygen species of O^2−^, O^−^, and O_2_^−^ oxygen adsorbed from the atmosphere to enhance the response [62]. Accordingly, it is taken for granted that the Eu-doped SnO_2_ shows a higher response than that of the pure.

Here, the mechanism as to the Eu-doped contents that greatly impacted on the response of sensors for methanal is discussed. As shown in Figure 8, for lower level of the Eu-doped SnO_2_ nanoarrays sensor, the response improved with the increase of the Eu-doped concentration. Consequently, the response of the 0.2 at % Eu-doped SnO_2_ nanoarrays is much better than the response of undoped thicker nanorods arrays, while the response of the 0.5 at % Eu-doped SnO_2_ nanoarrays sensor is higher than that of the undoped nanoarrays sensor and the 0.2 at % Eu-doped SnO_2_ nanoarrays sensor. However, the tendency of response declines when the Eu-doped content increases to more than 0.5 at %. For the Eu-doped contents of the 1.0, 1.5, and 3.0 at %, the responses of Eu-doped SnO_2_ nanoarrays sensors are 25, 23, and 21, respectively. It indicates that the response does not increase with the Eu-doped content increases. The possible reason is that redundant Eu doping will cause the formation of second phases, such as Eu_2_Sn_2_O_7_ or Eu_2_O_3_ (as shown in the XRD results) on the SnO_2_ nanoarrays [37], which decrease the electric transduction of the SnO_2_ nanoarrays. Thus, it would hamper the response of the SnO_2_ nanoarrays sensors to improve the gas-sensing properties. On the other hand, due to the Eu dopant as one of high-activity catalysts, methanal molecules could be burned on Eu_2_O_3_ or Eu_2_Sn_2_O_7_ crystalline grains and transformed into CO_2_ and H_2_O without producing any electric signals. Hence, only just an appropriate Eu-doped content is propitious to improve the SnO_2_ gas-sensing properties greatly.

Moreover, the excellent catalytic performance of Eu oxide, such as Eu_2_O_3_ or Eu_2_Sn_2_O_7_, needs to be taken into account. As one of the high-activity catalysts, Eu oxide can heighten the reactivity of the reaction between the methanal molecules and the oxygen species on the surface of the SnO_2_ to promote the decomposition of the detected gases. Thus, Eu dopant can also serve as sensitizers to increase the surface active sites, which are attributed to improve the response of the SnO_2_ arrays sensors. In a word, although it is complicated that the enhancement of ordered SnO_2_ nanorods arrays doped by Eu and need to be further study, the mechanism can be explained by the aspects above disscused.

## 4. Conclusions

In summary, a facile hydrothermal method without any surfactants and the absence of substrates was adopted to synthesize different Eu-doped levels of the SnO_2_ nanorods arrays. SEM survey results of the Eu-doped demonstrated a unique layered SnO_2_ nanoarray when combined by SnO_2_ nanorods with a diameter of 10 nm and length of several hundred nanometers. Eu dopant acts as a crystalline growth inhibitor to prevent the SnO_2_ nanorods growth up and obtain tenuous SnO_2_ nanorods arrays. X-ray diffraction (XRD) patterns recorded from all of the samples revealed the tetragonal rutile-type structure with a systematic average size reduction, while enhancing the residual strain (in the range of 0.1924% to 0.2794%) as the Eu-content increases. Transmission electron micrographs revealed the surface defects of crystal structure caused by the Eu ions doped into the SnO_2_ lattice. The Eu doping can greatly improve the gas sensing properties. The Eu-doped SnO_2_ layered nanorods arrays with an optimized Eu doping level of 0.5 at % exhibited an excellent sensing response toward methanal at a lower temperature of 278 °C. The Eu-doped SnO_2_ nanoarray is a promising sensing-material for trace methanal detection in environmental gas monitor.

## Figures and Tables

**Table 1:** List of crystallite size, residual strain, lattice parameters obtained from the XRD data of the layered Eu-doped SnO_2_ nanorods arrays.

Eu (*x*, at %) (Nominal)	Mean Crystallite Size (*D*) (nm)	Residual Strain (*ε*) (%)	Lattice Parameters (Å)		*c*/*a* Ratio	*u* (Å)	Unit Cell Volume (*V*/Å^3^)
			*a* (Å)	*c* (Å)			
0	26.7	0.1924	4.7363	3.1852	0.6725	0.3014	71.45
0.2	14.5	0.2369	4.7391	3.1895	0.6730	0.2998	71.63
0.5	8.8	0.2682	4.7428	3.1961	0.6739	0.2991	71.89
1.0	9.7	0.2719	4.7432	3.1992	0.6745	0.2936	71.98
1.5	12.3	0.2769	4.7436	3.1984	0.6743	0.2965	71.97
3.0	16.6	0.2794	4.7439	3.1997	0.6744	0.2994	72.01
